# Sub-Sharvin Conductance and Incoherent Shot-Noise in Graphene Disks at Magnetic Field

**DOI:** 10.3390/ma17133067

**Published:** 2024-06-21

**Authors:** Adam Rycerz, Katarzyna Rycerz, Piotr Witkowski

**Affiliations:** 1 Institute for Theoretical Physics, Jagiellonian University, Łojasiewicza 11, PL-30348 Kraków, Poland; pm.witkowski@uj.edu.pl; 2 Faculty of Computer Science, AGH University of Krakow, al. Mickiewicza 30, PL-30059 Kraków, Poland

**Keywords:** graphene, shot noise, Corbino disk, Landauer–Büttiker formalism

## Abstract

Highly doped graphene samples show reduced conductance and enhanced shot-noise power compared with standard ballistic systems in two-dimensional electron gas. These features can be understood within a model that assumes incoherent scattering of Dirac electrons between two interfaces separating the sample and the leads. Here we find, by adopting the above model for the edge-free (Corbino) geometry and by computer simulation of quantum transport, that another graphene-specific feature should be observable when the current flow through a doped disk is blocked by a strong magnetic field. When the conductance drops to zero, the Fano factor approaches the value of F≈0.56, with a very weak dependence on the ratio of the disk radii. The role of finite source-drain voltages and the system behavior when the electrostatic potential barrier is tuned from a rectangular to a parabolic shape are also discussed.

## 1. Introduction

Although the electronic properties of matter are governed by the rules of quantum mechanics [1], it is very unlikely to find that any measurable characteristic of a macroscopic system is determined solely by the universal constants of nature, such as the elementary charge (*e*) or the Planck constant (*h*). In the last century, two notable exceptions arrived with the phenomena of superconductivity [2], namely, the quantization of the magnetic flux piercing the superconducting circuit, which is the multiplicity of the flux quantum $\Phi_{0}=h/\left(2e\right)$ [3,4], and the *ac* Josephson effect, with the universal frequency-to-voltage ratio given by $2e/h=1/\Phi_{0}$ [5]. Later, with the advent of semiconducting heterostructures [6], came the quantum Hall effect [7,8,9,10,11,12] and the conductance quantization [13], bringing us the conductance quantum $g_{0}=se^{2}/h$ (with the degeneracy s=1, 2, or 4). The further development of nanosystems led to the observation of the Aharonov–Bohm effect, which is manifested by magnetoconductance oscillations with the period $2\Phi_{0}=h/e$ [14], as well as the universal conductance fluctuations [15,16,17,18], characterized by a variance $\propto \beta^{-1}{\left(se^{2}/h\right)}^{2}$, with an additional symmetry-dependent prefactor (β=1, 2, or 4). A related but slightly different issue concerns the Wiedemann–Franz (WF) law, which defines the Lorentz number, $L_{0}=\frac{\pi^{2}}{3}{\left(k_{B}/e\right)}^{2}$ (with the Boltzmann constant $k_{B}$) [2], as the proportionality coefficient between the electronic part of the thermal conductivity and the electrical conductivity multiplied by the absolute temperature. Although the WF law is obeyed with a few percent accuracy in various condensed matter systems it has never been shown to have metrological accuracy [19,20,21,22,23,24].

Some new ‘magic numbers’ similar to those mentioned above have arrived with the discovery of graphene, an atomically thin form of carbon [11,12]. For undoped graphene samples, charge transport is dominated by transport via evanescent modes [25], resulting in the universal *dc* conductivity $4e^{2}/\left(\pi h\right)$ accompanied by the sub-Poissonian shot noise, with a Fano factor F=1/3 [26,27,28,29,30,31]. For high frequencies, the *ac* conductivity is given by $\pi e^{2}/\left(2h\right)$, which leads to the quantized visible-light opacity πα (where α≈1/137.036 is the fine-structure constant) [32,33,34]. A possible new universal value is predicted for the maximum absolute thermopower, which approaches $\approx k_{B}/e$ near the charge neutrality point, for both monolayer and gapless bilayer graphene [35,36,37,38,39].

**Figure 1:**
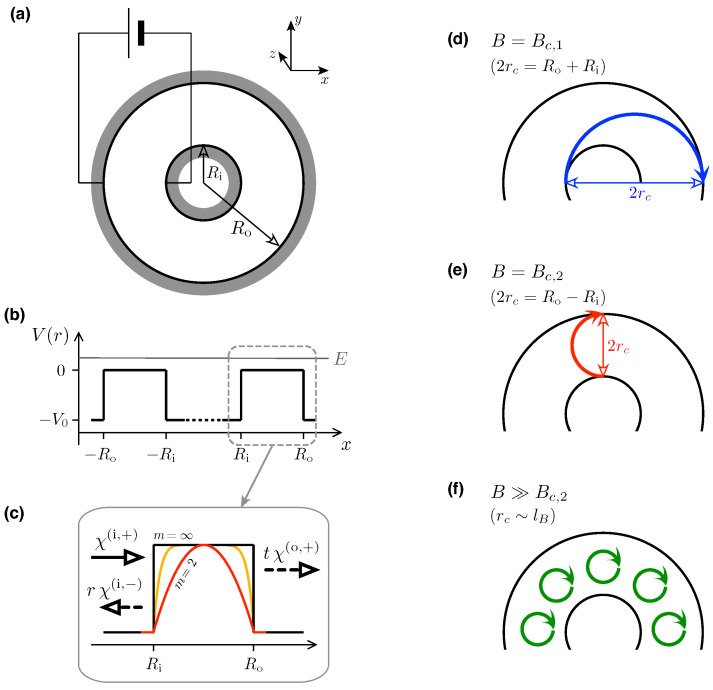
(**a**) Schematic of Corbino disk in graphene, with the inner radius, $R_{i}$, and the outer radius, $R_{o}$, contacted by two circular electrodes (dark areas). A voltage source drives a current through the disk. A separate gate electrode (not shown) allows us to tune the carrier concentration around the neutrality point. The coordinate system (x,y,z) is also shown. (**b**) Cross section of the electrostatic potential profile given by Equation (2) with m→∞ (i.e., the rectangular barrier) at y=z=0. (**c**) Zoom-in of a single barrier, for x>0, showing also the profiles for m=2 and 8, with symbolic representations of the incident and reflected waves in inner electrode ($x<R_{i}$) and the transmitted wave in outer electrode ($x>R_{o}$), with the amplitudes *r* and *t* corresponding to the Fermi energy E>0. (**d**–**f**) Characteristic values of the magnetic field B=(0,0,B) separating different transport regimes. At $B=B_{c,1}$, the cyclotron diameter $2r_{c}=R_{o}+R_{i}$, and the particle leaving the inner lead approaches the outer lead regardless of the initial direction (**d**). At $B=B_{c,2}$, we have $2r_{c}=R_{o}-R_{i}$, and only the trajectory tangent to the inner lead reaches the outer lead (**e**). For higher fields, classical trajectories do not contribute to the charge transport, which is possibly only if the resonance with the Landau level occurs for $E\approx E_{n\mathrm{LL}}$, with n=0,±1,±2,⋯ (**f**).

Away from the charge-neutrality point, ballistic graphene samples exhibit sub-Sharvin charge transport [40,41], characterized by a conductance reduced by a factor of π/4 compared with standard Sharvin contacts in two-dimensional electron gas (2DEG) [42,43]. What is more, the shot noise is enhanced (compared with 2DEG) up to F≈1/8 far from the charge-neutrality [30,31]. The detailed dependence of the above factors on the sample geometry has recently been discussed in analytical terms [41], using the example of an edge-free (Corbino) setup, characterized by the inner radius, $R_{i}$, and the outer radius, $R_{o}$ (see Figure 1). It is further found in Refs. [40,41] that the ballistic values of the conductance and the Fano factor are gradually restored when the potential barrier, which defines a sample area in the effective Dirac–Weyl Hamiltonian, evolves from a rectangular toward a parabolic shape.

Here we focus on the Corbino geometry, which is often considered when discussing fundamental aspects of graphene [25,44,45,46,47,48,49,50,51,52,53,54,55]. In this geometry, charge transport at high magnetic fields is unaffected by edge states, allowing bulk transport properties to be studied [48,49,50,51,52,53,54]. Recently, we have shown numerically that the thermoelectric properties in such a situation are determined by the energy interval separating consecutive Landau levels rather than by the transport gap (being the energy interval, for which the cyclotron diameter $2r_{c}<R_{o}-R_{i}$) [55]. In this paper, we address the question of how the shot noise behaves when the tunneling conductance regime is entered by increasing the magnetic field at a fixed doping (or decreasing the doping at a fixed field) such that $2r_{c}≲R_{o}-R_{i}$. Going beyond the linear-response regime, we find that the threshold voltage, $U_{\mathrm{on}}$, defined as the source-drain voltage difference that activates the current at minimum doping, is accompanied by the quasi-universal (i.e., weakly dependent on the radii ratio $R_{o}/R_{i}$) value of F≈0.56. The robustness of the effect is also analyzed when smoothing the electrostatic potential barrier.

The paper is organized as follows. In Section 2 we briefly present the effective Dirac Hamiltonian and the numerical approach used in the remaining parts of the paper. In Section 3, we derive an approximation for the transmission through a doped Corbino disk at non-zero magnetic field and subsequent formulas for the charge-transfer characteristics: the conductance and the Fano factor. Our numerical results, for both the rectangular and the smooth potential barriers, are presented in Section 4. The conclusions are given in Section 5.

## 2. Model and Methods

### 2.1. Dirac Equation for the Disk Geometry

Our analysis of the device schematically shown in Figure 1 starts from the effective wave equation for Dirac fermions in graphene, near the *K* valley,

(1) $$\left[v_{F}\;\left(p+eA\right)\cdot \sigma +V\left(r\right)\right]\Psi =E\Psi ,$$

where the Fermi velocity is given by $v_{F}=\sqrt{3}\;t_{0}a/\left(2\hbar \right)$, with $t_{0}=2.7\;$eV the nearest-neighbor hopping integral and a=0.246nm the lattice parameter, $p=-i\hbar \;(\partial_{x},\partial_{y})$ is the in-plane momentum operator, and we choose the symmetric gauge $A=\frac{B}{2}\left(-y,x\right)$ corresponding to the perpendicular, uniform magnetic field B=(0,0,B), and $\sigma =(\sigma_{x},\sigma_{y})$, where $\sigma_{j}$ are the Pauli matrices. For the forthcoming numerical calculations, we set (in the physical units) $\hbar v_{F}=0.575214\;$eV·nm, and e/(πℏ)=2067.83T^−1^·nm^−2^. The electrostatic potential energy in Equation (1), V(r), is given by

(2) $$V\left(r\right)=-V_{0}\times \left\{\begin{array}{cc} \;\frac{2^{m}{\left|r-R_{\mathrm{av}}\right|}^{m}}{|R_{o}-R_{i}{|}^{m}}\; & \mathrm{if}\;\;|r\;-\;R_{\mathrm{av}}|⩽\frac{R_{o}-R_{i}}{2},\\ \;1 & \mathrm{if}\;\;|r\;-\;R_{\mathrm{av}}|>\frac{R_{o}-R_{i}}{2}, \end{array}\right.$$

where we have defined $R_{\mathrm{av}}=\left(R_{i}+R_{o}\right)/2$. In particular, the limit of m→∞ corresponds to the rectangular barrier (with a cylindrical symmetry); any finite m⩾2 defines a smooth potential barrier, interpolating between the parabolic (m=2) and rectangular shapes. In principle, barrier smoothing can be regarded as a feature of a self-consistent solution resulting from carrier diffusion; we expect this feature to be strongly dependent on the experimental details, with graphene-on-hBN devices [49] showing rectangular rather than smooth profiles.

The symmetry of the problem allows one to search for the wave function in the form

(3) $$\Psi_{j}\left(r,\varphi \right)=e^{i(j-1/2)\varphi }\left(\begin{array}{c} \chi_{a}\\ \chi_{b}e^{i\varphi } \end{array}\right),$$

where j=±1/2,±3/2,⋯ is the total angular-momentum quantum number, the components $\chi_{a}=\chi_{a}\left(r\right)$, $\chi_{b}=\chi_{b}\left(r\right)$, and we have introduced the polar coordinates (r,φ). Substituting the above into Equation (1) brings us to the system of ordinary differential equations

(4) $$\begin{array}{cc} \chi_{a}^{\prime } & =\left(\frac{j-1/2}{r}+\frac{eBr}{2\hbar }\right)\chi_{a}+i\;\frac{E-V(r)}{\hbar v_{F}}\chi_{b}, \end{array}$$

(5) $$\begin{array}{cc} \chi_{b}^{\prime } & =i\;\frac{E-V(r)}{\hbar v_{F}}\chi_{a}-\left(\frac{j+1/2}{r}+\frac{eBr}{2\hbar }\right)\chi_{b}, \end{array}$$

where primes denote derivatives with respect to *r*.

### 2.2. Analytic Solutions

For the disk area, $R_{i}<r<R_{o}$, Equations (4) and (5) must typically be integrated numerically; key details of the procedure are presented in Appendix A. Here we focus on the special case of the rectangular barrier (m=∞), for which some analytical solutions have been reported [44,45,56].

In particular, in the absence of a magnetic field (B=0), the spinors $\chi_{j}={\left(\chi_{a},\chi_{b}\right)}^{T}$ corresponding to different *j*-s can be written as linear combinations [44]

(6) $$\chi_{j}^{\left(\mathrm{disk}\right)}=A_{j}\left(\begin{array}{c} H_{j-1/2}^{\left(2\right)}\left(kr\right)\\ i\eta H_{j+1/2}^{\left(2\right)}\left(kr\right) \end{array}\right)+B_{j}\left(\begin{array}{c} H_{j-1/2}^{\left(1\right)}\left(kr\right)\\ i\eta H_{j+1/2}^{\left(1\right)}\left(kr\right) \end{array}\right),$$

where $H_{\nu }^{\left(1\right)}\left(\rho \right)$ [$H_{\nu }^{\left(2\right)}\left(\rho \right)$] is the Hankel function of the first [second] kind, $k=|E|/(\hbar v_{F})$, the doping sign η=sgnE=±1 (with η=+1 indicating electron doping and η=−1 indicating hole doping), and $A_{j}$, $B_{j}$ are arbitrary complex coefficients. For B>0, Equation (6) is replaced by [45,56]

(7) $$\chi_{j}^{\left(\mathrm{disk}\right)}=A_{j}\left(\begin{array}{c} \xi_{j↑}^{\left(1\right)}\\ i\eta z_{j,1}\xi_{j↓}^{\left(1\right)} \end{array}\right)+B_{j}\left(\begin{array}{c} \xi_{j↑}^{\left(2\right)}\\ i\eta z_{j,2}\xi_{j↓}^{\left(2\right)} \end{array}\right),$$

where $z_{j,1}={\left[2\left(j+s_{j}\right)\right]}^{-2s_{j}}$, $z_{j,2}=2{\left(\beta /k^{2}\right)}^{s_{j}+1/2}$ (with $s_{j}\equiv \frac{1}{2}sgn\;j$, β=eB/(2ℏ)), and

(8) $$\xi_{js}^{\left(\nu \right)}=e^{-\beta r^{2}/2}{\left(kr\right)}^{|l_{s}|}\left\{\begin{array}{cc} M(\alpha_{js},\gamma_{js},\beta r^{2}), & \nu \;=\;1,\\ U(\alpha_{js},\gamma_{js},\beta r^{2}), & \nu \;=\;2, \end{array}\right.$$

with $l_{s}=j\mp \frac{1}{2}$ for s=↑,↓, $\alpha_{js}=\frac{1}{4}[2(l_{-s}+|l_{s}|+1)-k^{2}/\beta ]$, and $\gamma_{js}=\left|l_{s}\right|+1$. M(a,b,z) and U(a,b,z) are the confluent hypergeometric functions [57].

For the leads, $r<R_{i}$ or $r>R_{o}$, the electrostatic potential energy is constant, $V\left(r\right)=-V_{0}$. We further assume B=0 and $E>-V_{0}$ (electron doping) in the leads, which allows us to adapt the wave function given by Equation (6); i.e., for the inner lead, $r<R_{i}$,

(9) $$\chi_{j}^{\left(\mathrm{inner}\right)}=\left(\begin{array}{c} H_{j-1/2}^{\left(2\right)}\left(Kr\right)\\ iH_{j+1/2}^{\left(2\right)}\left(Kr\right) \end{array}\right)+r_{j}\left(\begin{array}{c} H_{j-1/2}^{\left(1\right)}\left(Kr\right)\\ iH_{j+1/2}^{\left(1\right)}\left(Kr\right) \end{array}\right),$$

and for the outer lead $r>R_{o}$,

(10) $$\chi_{j}^{\left(\mathrm{outer}\right)}=t_{j}\left(\begin{array}{c} H_{j-1/2}^{\left(2\right)}\left(Kr\right)\\ iH_{j+1/2}^{\left(2\right)}\left(Kr\right) \end{array}\right),$$

where $K=|E+V_{0}|/\left(\hbar v_{F}\right)$, and we have introduced the reflection and transmission coefficient. The first spinor in each of Equations (9) and (10) represents the incoming (i.e., propagating from r=0) wave, and the second spinor in Equation (9) represents the outgoing (propagating from r=∞) wave.

### 2.3. Mode-Matching Method

Since the current-density operator following from Equation (1), $j=ev_{F}\sigma$, does not involve differentiation, the mode-matching conditions for $r=R_{i}$ and $r=R_{o}$ reduce to the equalities for spinor components, namely

(11) $$\chi_{j}^{\left(\mathrm{inner}\right)}\left(R_{o}\right)=\chi_{j}^{\left(\mathrm{disk}\right)}\left(R_{o}\right)\;\;\mathrm{and}\;\;\chi_{j}^{\left(\mathrm{disk}\right)}\left(R_{i}\right)=\chi_{j}^{\left(\mathrm{outer}\right)}\left(R_{i}\right).$$

The resulting formula for the transmission probability for the *j*-th mode becomes particularly simple if we consider the limit of heavily doped leads, $U_{0}\to \infty$. In particular, for B=0, substituting Equation (6) into the above gives [58]

(12) $$T_{j}={\left|t_{j}\right|}^{2}=\frac{16}{\pi^{2}k^{2}R_{i}R_{o}}\;\frac{1}{{\left[D_{j}^{\left(+\right)}\right]}^{2}+{\left[D_{j}^{\left(-\right)}\right]}^{2}},$$

where

(13) Dj(±)=ImHj−1/2(1)(kRi)Hj∓1/2(2)(kRo)±Hj+1/2(1)(kRi)Hj±1/2(2)(kRo).

Probably, the most surprising feature of the above result is that taking the limit of $U_{0}\to \infty$ does not give $T_{j}\to 0$ for all *j*-s; instead, there is a set of $T_{j}∼1$ for $\left|j\right|≲kR_{i}$. (The corresponding discussion for the Corbino disk in 2DEG can be found in Ref. [44].)

Analogously, for B>0 one finds, using Equations (7) and (8),

(14) $$T_{j}={\left|t_{j}\right|}^{2}=\frac{16\;{\left(k^{2}/\beta \right)}^{\left|2j-1\right|}}{k^{2}R_{i}R_{o}\;\left(X_{j}^{2}+Y_{j}^{2}\right)}{\left[\frac{\Gamma (\gamma_{j↑})}{\Gamma (\alpha_{j↑})}\right]}^{2},$$

where Γ(z) is the Euler Gamma function, and

(15) $$\begin{array}{cc} \; & X_{j}=w_{j↑↑}^{-}+z_{j,1}z_{j,2}w_{j↓↓}^{-},\;\;\;Y_{j}=z_{j,2}w_{j↑↓}^{+}-z_{j,1}w_{j↓↑}^{+},\\ \; & w_{jss^{\prime }}^{\pm }=\xi_{js}^{\left(1\right)}\left(R_{i}\right)\xi_{js^{\prime }}^{\left(2\right)}\left(R_{o}\right)\pm \xi_{js}^{\left(1\right)}\left(R_{o}\right)\xi_{js^{\prime }}^{\left(2\right)}\left(R_{i}\right). \end{array}$$

For B<0, one obtains $T_{j}\left(B\right)=T_{-j}\left(-B\right)$.

Details of numerical mode-matching, applicable to smooth potentials, are given in Appendix A.

### 2.4. Landauer–Büttiker Formalism

If the nanoscopic system is connected to external reservoirs, characterized by the electrochemical potentials μ and $\mu +eU_{\mathrm{eff}}$ (for simplicity, the two reservoirs are considered; for a more general discussion, see Ref. [59]), the conductance of the system is related to the transmission probabilities for normal modes ($T_{j}$-s) by

(16) $$G\left(U_{\mathrm{eff}}\right)=\frac{\langle I\rangle }{U_{\mathrm{eff}}}=\frac{g_{0}}{U_{\mathrm{eff}}}\int_{\mu }^{\mu +eU_{\mathrm{eff}}}d\epsilon \sum_{j}T_{j}\left(\epsilon \right),$$

where 〈I〉 denotes the average electric current and the zero-temperature limit is taken. The conductance quantum is $g_{0}=4e^{2}/h$, taking into account spin and valley degeneracies. $U_{\mathrm{eff}}$ is the effective voltage difference between the reservoirs (note that the actual voltage applied may differ from $U_{\mathrm{eff}}$ due to charge-screening effects). Similarly, the Fano factor, which relates the current variance, $\langle {\left(I-\langle I\rangle \right)}^{2}\rangle$, to the value ${\langle {\left(I-\langle I\rangle \right)}^{2}\rangle }_{\mathrm{Poisson}}$, which one would measure in the absence of correlations between scattering events (occurring, e.g., in the tunneling limit of $T_{j}\ll 1$ for all *j*-s), is given by

(17) $$F\left(U_{\mathrm{eff}}\right)=\frac{\langle {\left(I-\langle I\rangle \right)}^{2}\rangle }{{\langle {\left(I-\langle I\rangle \right)}^{2}\rangle }_{\mathrm{Poisson}}}=\frac{g_{0}}{GU_{\mathrm{eff}}}\int_{\mu }^{\mu +eU_{\mathrm{eff}}}d\epsilon \sum_{j}T_{j}\left(\epsilon \right)\left[1-T_{j}\left(\epsilon \right)\right],$$

where ${\langle {\left(I-\langle I\rangle \right)}^{2}\rangle }_{\mathrm{Poisson}}=e\langle I\rangle /\Delta t=eGU_{\mathrm{eff}}/\Delta t$, with Δt denoting the time of the measurement.

For the sake of completeness, we emphasize here that the non-interacting Landauer–Büttiker formalism, as presented above, is valid for weak currents, when the system-reservoir distinction is possible. Generalizations for strong coupling [60,61], as well as for many-body correlated nanosystems [62], have recently been proposed. However, such situations are beyond the scope of the present work.

In the linear-response regime ($U_{\mathrm{eff}}\to 0$), Equations (16) and (17) reduce to

(18) $$G\left(U_{\mathrm{eff}}\to 0\right)=g_{0}\sum_{j}T_{j},$$

and

(19) $$F\left(U_{\mathrm{eff}}\to 0\right)=\frac{\sum_{j}T_{j}\left(1-T_{j}\right)}{\sum_{j}T_{j}},$$

where $T_{j}=T_{j}\left(\mu \right)$. For the disk geometry, the summation range is limited by the number of propagating modes in the inner lead, $\left|j\right|⩽j_{\max}=\left\lfloor KR_{i}\right\rfloor -\frac{1}{2}$ where ⌊x⌋ is the floor function of *x*. (For heavily doped leads, $j_{\max}\to \infty$.) Experimentally obtained conductance spectra for suspended graphene disks [48] show relatively good agreement with the formula following from Equations (12), (13), and (18), provided that an adjustable parameter, quantifying the contact resistance between the electrodes and the sample, is taken into account. To the best of our knowledge, noise measurements for the disk geometry are still missing.

As a notable example, let us consider the zero-doping limit (μ→0). In such a case, Equation (14) can be simplified as follows [45,63]

(20) $$T_{j}\left(\mu \to 0\right)=\frac{1}{\cosh^{2}\left[\left(j+\Phi /\Phi_{0}\right)\ln\left(R_{o}/R_{i}\right)\right]},$$

where $\Phi =\pi (R_{o}^{2}-R_{i}^{2})B$ is the flux piercing the disk area, and we have defined $\Phi_{0}=2\;\left(h/e\right)\ln\left(R_{o}/R_{i}\right)$. Assuming the narrow-disk range, $R_{o}\approx R_{i}$, we can approximate the sums occurring in Equations (18) and (19) by integrals, obtaining

(21) $$G\approx G_{\mathrm{diff}}=\frac{2\pi \sigma_{0}}{\ln(R_{o}/R_{i})}\;\;\;\;\mathrm{and}\;\;\;\;\;F\approx F_{\mathrm{diff}}=\frac{1}{3}.$$

The above reproduces the pseudodiffusive conductance and the shot-noise power for a disk geometry [44]. For larger $R_{o}/R_{i}$, both characteristics are predicted to show approximately sinusoidal conductance oscillations with the field *B* [45,63,64].

The case of the doped disk, for which one may expect to observe some features of the sub-Sharvin charge transport [40,41], is discussed next.

## 3. Approximate Conductance and Fano Factor at Magnetic Field

Before calculating the conductance, *G*, and the Fano factor, *F*, within the mode-matching method described in Section 2, we first present the approximation formulas for the incoherent transport, obtained by adapting the derivation of Ref. [41] for the B>0 case.

In short, the approximation is based on two key assumptions. (i) We neglect the evanescent waves, which are present in the exact solutions from which one obtains $T_{j}>0$ for arbitrarily large |j|, see Equations (12)–(15). In turn, zero linear-response conductance, see Equation (18), is expected for $B>B_{c,2}$. In such a range, the approximate Fano factor given by Equation (19) will be undefined (since the actual value of *F* is governed by evanescent waves); however, the limit of $B\to B_{c,2}-$ could still be taken (and is not necessarily determined by the evanescent contribution). In addition, (ii) the incoherent scattering is assumed, i.e., the phase gained by the wave propagating in the sample area is assumed to be random, eliminating the resonances with Landau levels present in the exact solution [55].

### 3.1. Corbino Disk in Graphene as a Double Barrier

**Figure 2:**
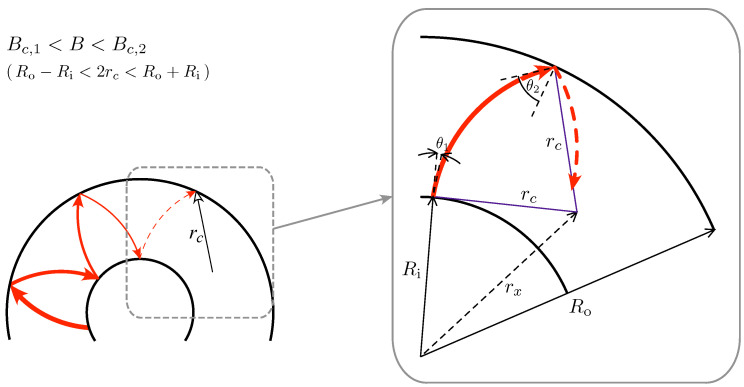
Propagation between consecutive scatterings (red lines with arrows) on interfaces at $r=R_{i}$ and $r=R_{o}$ in a uniform magnetic field $B_{c,1}<B<B_{c,2}$. A zoom-in showing an arc of single cyclotron orbit centered at $r=r_{x}$, with radius $r_{c}$, and incident angles $\theta_{1}$ (for $r=R_{i}$) and $\theta_{2}$ (for $r=R_{o}$).

A key step in the derivation is the observation that in the multimode regime ($kR_{i}\gg 1$), for which one can consider well-defined trajectories, the disk symmetry causes the incident angles $\theta_{1}$ and $\theta_{2}$, corresponding to the interfaces at $r=R_{i}$ and $r=R_{o}$ (see Figure 2), to remain constant (up to the sign) after multiple scattering. Therefore, the double-contact formula for incoherent transmission [65,66] can be applied, namely

(22) $$\begin{array}{cc} {\left\{T\right\}}_{\mathrm{incoh}} & =\frac{1}{2\pi }\int_{-\pi }^{\pi }d\phi \;\frac{T_{1}T_{2}}{2-T_{1}-T_{2}+T_{1}T_{2}-2\sqrt{\left(1-T_{1}\right)\left(1-T_{2}\right)}\cos\phi }\\ \; & =\frac{T_{1}T_{2}}{T_{1}+T_{2}-T_{1}T_{2}}, \end{array}$$

where the transmission probabilities $T_{1}$, $T_{2}$, corresponding to a potential step of infinite height, are given by

(23) $$T_{l}=\frac{2\cos\theta_{l}}{1+\cos\theta_{l}},\;\;\;\;\;\;l=1,2,$$

and ϕ is assumed to be a random phase acquired during the propagation between $r=R_{i}$ and $r=R_{o}$ (or vice versa). Similarly, we calculate the incoherent squared transmission, which is useful when evaluating the Fano factor,

(24) $$\begin{array}{cc} {\left\{T^{2}\right\}}_{\mathrm{incoh}} & =\frac{1}{2\pi }\int_{-\pi }^{\pi }d\phi \;{\left(\frac{T_{1}T_{2}}{2-T_{1}-T_{2}+T_{1}T_{2}-2\sqrt{\left(1-T_{1}\right)\left(1-T_{2}\right)}\cos\phi }\right)}^{2}\\ \; & =\frac{{\left(T_{1}T_{2}\right)}^{2}\left(2-T_{1}-T_{2}+T_{1}T_{2}\right)}{{\left(1+T_{1}T_{2}-T_{1}T_{2}\right)}^{3}}. \end{array}$$

Next, the incoherent conductance in the linear-response regime is evaluated by inserting ${\left\{T\right\}}_{\mathrm{incoh}}$ (22) into Equation (18),

(25) $$G_{\mathrm{incoh}}=G_{\mathrm{Sharvin}}{\langle {\left\{T\right\}}_{\mathrm{incoh}}\rangle }_{u=\sin\theta_{1}},$$

with

(26) $$G_{\mathrm{Sharvin}}=2g_{0}kR_{i}.$$

By analogy, for the Fano factor we can derive the following from Equation (19)

(27) $$F_{\mathrm{incoh}}=1-\frac{{\langle {\left\{T^{2}\right\}}_{\mathrm{incoh}}\rangle }_{u=\sin\theta_{1}}}{{\langle {\left\{T\right\}}_{\mathrm{incoh}}\rangle }_{u=\sin\theta_{1}}}.$$

The summation over $2kR_{i}$ modes is approximated in Equations (25) and (27) by averaging over the variable $u=\sin\theta_{1}$, in the range of −1⩽u⩽1. Explicitly,

(28) $${\langle {\left\{T^{n}\right\}}_{\mathrm{incoh}}\rangle }_{u=\sin\theta_{1}}=\frac{1}{2}\int_{u_{c}}^{1}du{\left\{T^{n}\right\}}_{\mathrm{incoh}},\;\;\;\;n=1,2,$$

where the lower integration limit ($u_{c}$) is defined by the value of $\sin\theta_{1}$, below which the trajectory cannot reach the outer interface ($r=R_{o}$). (In other words, for $u=\sin\theta_{1}<u_{c}$, the geometric derivation below yields $|\sin\theta_{2}|>1$).

The missing elements, necessary to calculate ${\langle {\left\{T^{n}\right\}}_{\mathrm{incoh}}\rangle }_{u=\sin\theta_{1}}$ in Equation (28), are the dependence of $\theta_{2}$ on $\theta_{1}$ and *B* (see Equations (22)–(24)), as well as the dependence of $u_{c}$ on *B*.

Since we have assumed constant electrostatic potential energy in the disk area, the trajectory between successive scatterings (see Figure 2) forms an arc, with the constant radius

(29) $$r_{c}=\hbar k/\left(eB\right)=\left|E\right|/\left(v_{F}eB\right),$$

(i.e., the cyclotron radius for a massless Dirac particle at B>0), centered at the distance $r_{x}$ from the origin. Now, solving the two triangles with a common edge, $r_{x}$ (dashed line) and the opposite vertices in two scattering points, we find

(30) $$r_{x}^{2}=R_{i}^{2}+r_{c}^{2}+2R_{i}r_{c}\sin\theta_{1}$$

(for the triangle containing a scattering point at $r=R_{i}$), and

(31) $$r_{x}^{2}=R_{o}^{2}+r_{c}^{2}-2R_{o}r_{c}\sin\theta_{2}$$

(for the triangle containing the scattering point at $r=R_{o}$). Together, Equations (30) and (31) lead to

(32) $$\sin\theta_{2}=\frac{R_{o}^{2}-R_{i}^{2}-2R_{i}r_{c}u}{2R_{o}r_{c}}.$$

Subsequently, the value of $u_{c}$ in Equation (28) is given by

(33) $$u_{c}=\left\{\begin{array}{cc} -1, & \mathrm{if}\;\;B⩽B_{c,1}\\ \frac{R_{o}^{2}-R_{i}^{2}}{2R_{i}r_{c}}-\frac{R_{o}}{R_{i}},\; & \mathrm{if}\;\;B_{c,1}<B⩽B_{c,2}\\ 1, & \mathrm{if}\;\;B>B_{c,2} \end{array}\right.,$$

where we have additionally defined

(34) $$B_{c,m}=\frac{2\hbar k}{e\left[R_{o}-{\left(-1\right)}^{m}R_{i}\right]},\;\;\;\;\;m=1,2.$$

### 3.2. The Zero-Field Limit

Typically, averages occurring in Equations (25) and (27) must be evaluated numerically. Analytical expressions are available, e.g., for zero magnetic field [41]

(35) $$\begin{array}{cc} G_{\mathrm{incoh}}\left(B\;\to \;0\right) & =G_{\mathrm{Sharvin}}\;\frac{\left(2a+\frac{1}{a}\right)\arcsin a+3\sqrt{1-a^{2}}-\frac{\pi }{2}\left(a^{2}+2\right)}{1-a^{2}},\\ F_{\mathrm{incoh}}\left(B\;\to \;0\right) & =\left\{2a\sqrt{1-a^{2}}\left(53+279a^{2}+88a^{4}\right)-3\pi a\left(12+82a^{2}+45a^{4}+a^{6}\right)\right.\\ \; & \left.\;+6(1+45a^{2}+82a^{4}+12a^{6})\arcsin a\right\} \end{array}$$

(36) $$\begin{array}{cc} \; & /\left\{6{\left(1-a^{2}\right)}^{2}\left[\pi a\left(a^{2}+2\right)-6a\sqrt{1-a^{2}}-2\left(2a^{2}+1\right)\arcsin a\right]\right\}, \end{array}$$

where we have defined the inverse radii ratio $a=R_{i}/R_{o}$.

### 3.3. The Zero-Conductance Limit

In the this paper, we focus on the limit of $B\to B_{c,2}-$ (i.e., *B* approaching $B_{c,2}$ from below), for which $G_{\mathrm{incoh}}\to 0$. By introducing the dimensionless 0<ε≪1, one can express the cyclotron diameter, see Equation (29), as

(37) $$2r_{c}=R_{o}-R_{i}+\varepsilon \left(R_{o}-R_{i}\right).$$

In turn, the value $u_{c}$, see Equation (33), can be approximated (up to the leading order in ε) as follows

(38) $$u_{c}\approx 1-\varepsilon \left(1+\frac{R_{o}}{R_{i}}\right).$$

It is now convenient to define the variable

(39) $$\alpha =\frac{1-u}{\varepsilon \left(1+R_{o}/R_{i}\right)},$$

so that the integration over $u_{c}⩽u⩽1$, which occurs when evaluating ${\langle {\left\{T^{n}\right\}}_{\mathrm{incoh}}\rangle }_{u=\sin\theta_{1}}$ from Equation (28), can be replaced by an integration over 1⩾α⩾0. The transmission probabilities ($T_{1}$, $T_{2}$) for the interfaces at $r=R_{i}$ and $r=R_{o}$, see Equations (22)–(24) and (32), can now be approximated as

(40) $$\begin{array}{cc} T_{1} & \approx 2\sqrt{2\alpha \left(1+\frac{1}{a}\right)}\;\varepsilon^{1/2}, \end{array}$$

(41) $$\begin{array}{cc} T_{2} & \approx 2\sqrt{2\left(1-\alpha \right)\left(1+a\right)}\;\varepsilon^{1/2}, \end{array}$$

where we have used $a=R_{i}/R_{o}$ again.

Using the above expressions, we can now rewrite the averages in Equation (28), up to the leading order in ε, as follows

(42) $$\begin{array}{cc} {\langle {\left\{T\right\}}_{\mathrm{incoh}}\rangle }_{u} & =\frac{G_{\mathrm{incoh}}}{G_{\mathrm{Sharvin}}}\approx \frac{2\left(1+\frac{1}{a}\right)}{\sqrt{2}}\;\varepsilon^{3/2}\int_{0}^{1}d\alpha \frac{\sqrt{\alpha (1+\frac{1}{a})}\sqrt{\left(1-\alpha )(1+a\right)}}{\sqrt{\alpha (1+\frac{1}{a})}+\sqrt{\left(1-\alpha )(1+a\right)}}, \end{array}$$

(43) $$\begin{array}{cc} {\langle {\left\{T^{2}\right\}}_{\mathrm{incoh}}\rangle }_{u} & \approx \frac{2\left(1+\frac{1}{a}\right)}{\sqrt{2}}\;\varepsilon^{3/2}\int_{0}^{1}d\alpha \frac{\alpha \left(1-\alpha \right)\left(2+\frac{1}{a}+a\right)}{{\left[\sqrt{\alpha (1+\frac{1}{a})}+\sqrt{\left(1-\alpha )(1+a\right)}\right]}^{3}}. \end{array}$$

Remarkably, both quantities decay as $\propto \varepsilon^{3/2}$, but their ratio, which appears in Equation (27) for the Fano factor, remains constant (for a given *a*). The integrals in Equations (42) and (43) can be calculated analytically, resulting in

(44) $$\begin{array}{cc} F_{\mathrm{incoh}}\left(B\;\to \;B_{c,2}-\right) & =1-\left\{4\sqrt{a}+39a-58a^{3/2}-23a^{2}-23a^{5/2}-58a^{3}+39a^{7/2}+4a^{4}\right.\\ \; & \;+\left.\left(69a^{2}-18a^{3}-18a\right)\sqrt{1+a}\;artanh\left(\frac{\sqrt{a}}{\sqrt{1+a}}\right)\right.\\ \; & \;+\left.\left(69a^{2}-18a-18a^{3}\right)\sqrt{1+a}\;artanh\left(\frac{1}{\sqrt{1+a}}\right)\right\}\\ \; & \;/\left\{{\left(1+a\right)}^{3}\left(1+\sqrt{a}\right)\left(1-3\sqrt{a}+a\right)+3a{\left(1+a\right)}^{5/2}\;artanh\left[\frac{\left(1+\sqrt{a}\right)\sqrt{1+a}}{1+\sqrt{a}+a}\right]\right\}. \end{array}$$

In particular, for a→1, which represents the narrow-disk limit of $R_{o}\approx R_{i}$, the above reduces to

(45) $$F_{\mathrm{incoh}}{\left(B\;\to \;B_{c,2}-\right)}_{a\to 1}=1+\frac{38-33\sqrt{2}\;\mathrm{artanh}\left(\frac{1}{2}\sqrt{2}\right)}{6\sqrt{2}\;\mathrm{artanh}\left(\frac{2}{3}\sqrt{2}\right)-8}\approx 0.549708.$$

Numerical values of $F_{\mathrm{incoh}}\left(B\;\to \;0\right)$ and $F_{\mathrm{incoh}}\left(B\;\to \;B_{c,2}-\right)$ for selected $a=R_{i}/R_{o}$ are given in Table 1. For $F_{\mathrm{incoh}}\left(B\;\to \;B_{c,2}-\right)$, we see that the Poissonian value of $F_{\mathrm{incoh}}=1$, which could be expected due to the vanishing conductance, is reconstructed only for a→0 (i.e., for $R_{o}\gg R_{i}$). For finite radii ratios, non-trivial values of $0<F_{\mathrm{incoh}}<1$ occur. Remarkably, for moderate disk proportions (a⩾0.5), $F_{\mathrm{incoh}}\left(B\;\to \;B_{c,2}-\right)$ shows very weak dependence on *a*, decaying by less than 2% (from $F_{\mathrm{incoh}}\approx 0.56$ at a=0.5 to $F_{\mathrm{incoh}}\approx 0.55$ for a→1).

For this reason, in the following numerical analysis, we fixed the disk radii ratio at a=0.5 (i.e., $R_{o}=2R_{i}$). We also emphasize that the above derivation holds true for the parameter ε→0+, which quantifies the ratio of the cyclotron diameter, $2r_{c}$, to the radius difference, $R_{o}-R_{i}$, see Equation (37). Therefore, it is irrelevant whether one increases the magnetic field at a fixed chemical potential, or decreases the chemical potential at a fixed B>0 (as long as the system stays in a multimode range, $kR_{i}\gg 1$).

## 4. Results and Discussion

The main doubt that arises when we consider the applicability of Equation (44) to real quantum systems concerns the possible role of evanescent waves, which are completely neglected in our derivation. Obviously, they should not play an important role when the system is highly conducting (such as in the zero-field case [41]); however, since the Fano factor is determined by the ratio of two cumulants, both of which vanish for sufficiently high fields, it is not entirely clear which contribution (from propagating or from evanescent modes) would determine the value of *F* for $B\to B_{c,2}$. On the other hand, resonances with Landau levels are not expected to play a significant role, as they form very narrow transmission peaks whose contributions are immediately smeared out beyond the linear-response regime.

In the remaining parts of the paper, we compare the results of the computer simulation of quantum transport through the disk in graphene with the predictions for incoherent scattering presented in Section 3, in an attempt to propose an experimental procedure that allows one to extract the nontrivial value of F≈0.56 from the data plagued by other contributions.

### 4.1. The Rectangular Barrier of an Infinite Height

As a first numerical example, we took the limit of $V_{0}\to \infty$ and m→∞ in Equation (2), for which close-form expressions for the transmission probabilities were presented in Section 2.

**Figure 3:**
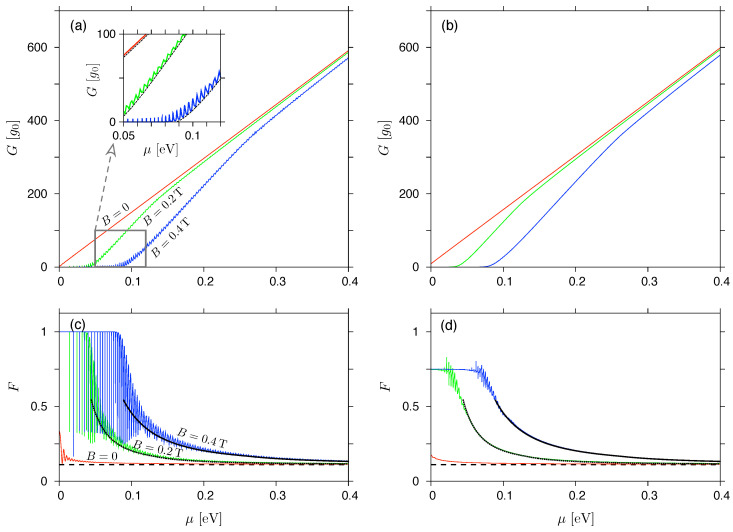
(**a**,**b**) Conductance and (**c**,**d**) the Fano factor for the Corbino disk in graphene with the radii $R_{o}=2R_{i}=1000\;$nm, and the rectangular potential barrier (i.e., $V_{0}\to \infty$ and m→∞ in Equation (2)) displayed as functions of the chemical potential. The values of magnetic field are B=0 (red solid lines in all plots), B=0.2T (green solid lines), and B=0.4T (blue solid lines). Inset in (**a**) is a zoom-in, with black dashed lines depicting the incoherent conductance, see Equation (25). (**a**,**c**) show the linear-response results, see Equations (18) and (19); the datasets in (**b**,**d**) are obtained from Equations (16) and (17) with $U_{\mathrm{eff}}=0.01\;$V. Remaining lines in (**c**,**d**) (black solid, black dotted, and black dashed) mark the incoherent Fano factor, see Equation (27); the values of magnetic field are specified for lines in (**c**), and are the same in (**d**). (For B=0, horizontal lines mark $F_{\mathrm{incoh}}\left(B\to 0\right)=0.111074$, corresponding to $R_{o}=2R_{i}$, see Table 1).

In Figure 3, we compare the linear-response conductance, $G(U_{\mathrm{eff}}\to 0)$, see Equation (18), with $G(U_{\mathrm{eff}})$ calculated from Equation (16) for a small but non-zero value of $U_{\mathrm{eff}}=0.01\;$V, both shown as functions of the chemical potential. Also in Figure 3 the same comparison is presented for the Fano factor, $F(U_{\mathrm{eff}})$ (see Equations (19) and (17)). It is easy to see that the prominent aperiodic oscillations visible for both charge-transfer cumulants in the $U_{\mathrm{eff}}\to 0$ limit are significantly reduced, even for small $U_{\mathrm{eff}}>0$. In fact, for $U_{\mathrm{eff}}=0.01\;$V and B>0, the values of $F_{\mathrm{incoh}}$ calculated from Equation (27) (black lines) are closely followed by $F(U_{\mathrm{eff}})$ obtained from the numerical mode matching, as long as the former can be defined, i.e., for $B<B_{c,2}$ at a given μ. We also note that the value of μ for which $B\approx B_{c,2}$ and $F(U_{\mathrm{eff}})\approx 0.56$ is accompanied by $G\left(U_{\mathrm{eff}}\right)∼g_{0}$ (up to the order of magnitude). For smaller μ, such that $B>B_{c,2}$ and $F_{\mathrm{incoh}}$ is undefined, $F(U_{\mathrm{eff}})$ saturates near the value ≈0.75, apparently below the Poissonian limit of F=1.

**Figure 4:**
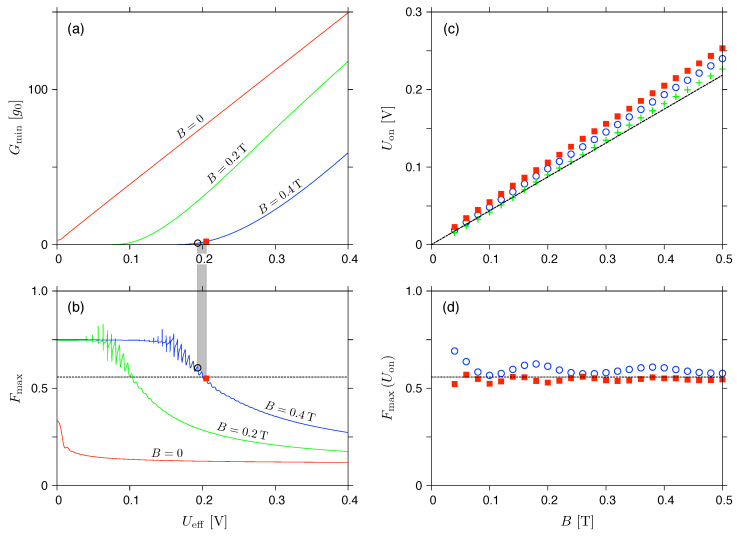
(**a**,**b**) Minimal conductivity ($G_{\min}$) and maximal Fano factor ($F_{\max}$), see Equations (16) and (17), corresponding to the chemical potential fixed at $\mu =-eU_{\mathrm{eff}}/2$, versus the effective voltage. The magnetic field (*B*) is specified for each line. (**c**) The activation voltage, defined via $G_{\min}\left(U_{\mathrm{on}}^{\left(1\right)}\right)=g_{0}$ (blue open circles), $G_{\min}\left(U_{\mathrm{on}}^{\left(2\right)}\right)=2g_{0}$ (red solid squares), or obtained from scaling according to Equation (47) (green crosses), displayed versus the magnetic field. (**d**) The Fano factor corresponding to $U_{\mathrm{eff}}=U_{\mathrm{on}}$ shown in (**c**). Horizontal dashed lines in (**b**,**d**) mark the value of $F_{\mathrm{incoh}}\left(B\to B_{c,2}-\right)=0.557898$ for $R_{o}=2R_{i}$ (see Table 1). Dashed line in (**c**) depicts the approximation given in Equation (46). The remaining system parameters are same as in Figure 3.

To better understand the nature of the results, we now (see Figure 4) go further beyond the linear-response regime, and calculate $G(U_{\mathrm{eff}})$ and $F(U_{\mathrm{eff}})$ for $\mu =-eU_{\mathrm{eff}}/2$ (note that for an infinite rectangular barrier we have the particle-hole symmetry, and both cumulants are even upon $\mu ↔-\mu +eU_{\mathrm{eff}}$), and display them as functions of $U_{\mathrm{eff}}$.

Next, we introduce the activation voltage $U_{\mathrm{on}}=U_{\mathrm{on}}\left(B\right)$, the meaning of which can be understood as follows. The cyclotron diameter, see Equation (29), naturally defines the range of energies for which $2r_{c}\left(E\right)<R_{o}-R_{i}$ and the system shows G≈0 (up to the evanescent modes). On the other hand, since we have set $\mu =-eU_{\mathrm{eff}}/2$, the effective voltage defines the energy range of $\left|E\right|⩽eU_{\mathrm{eff}}/2$, which is the integration interval in Equations (16) and (17). As a consequence, $G(U_{\mathrm{eff}})>0$ is expected for $U_{\mathrm{eff}}⩾U_{\mathrm{on}}$, which can be approximated as

(46) $$U_{\mathrm{on},\mathrm{incoh}}=v_{F}B\left(R_{o}-R_{i}\right),$$

where we have simply rewritten the equality $2r_{c}\left(eU_{\mathrm{on}}\right)=R_{o}-R_{i}$, neglecting the evanescent modes.

Looking at the conductance spectra shown in Figure 4a, we see for B>0 that a wide range of lower $U_{\mathrm{eff}}$, for which G≈0, is attached (via a cusp region) to the range of (approximately linearly) increasing *G*. To determine the value of $U_{\mathrm{on}}\left(B\right)$ directly from the conductance spectra, $G(U_{\mathrm{eff}})$, we numerically find the value of $U_{\mathrm{on}}^{\left(1\right)}$ such that $G\left(U_{\mathrm{on}}^{\left(1\right)}\right)=g_{0}$, and $U_{\mathrm{on}}^{\left(2\right)}$ such that $G\left(U_{\mathrm{on}}^{\left(2\right)}\right)=2g_{0}$, see the datapoints in Figure 4a. Then the linear extrapolation is performed to obtain

(47) $$\begin{array}{cc} U_{\mathrm{on}}^{\left(0\right)} & =U_{\mathrm{on}}^{\left(1\right)}-\left(U_{\mathrm{on}}^{\left(2\right)}-U_{\mathrm{on}}^{\left(1\right)}\right)\frac{G(U_{\mathrm{on}}^{\left(1\right)})}{G\left(U_{\mathrm{on}}^{\left(2\right)}\right)-G\left(U_{\mathrm{on}}^{\left(1\right)}\right)}\\ \; & =2U_{\mathrm{on}}^{\left(1\right)}-U_{\mathrm{on}}^{\left(2\right)}, \end{array}$$

so that $G(U_{\mathrm{on}}^{\left(0\right)})\approx 0$. The resulting values of $U_{\mathrm{on}}^{\left(i\right)}$, depicted in Figure 4c (datapoints), stay close to $U_{\mathrm{on},\mathrm{incoh}}$ given by Equation (46) (dashed line).

Remarkably, the values of the Fano factor corresponding to $U_{\mathrm{eff}}=U_{\mathrm{on}}^{\left(i\right)}$, i=1,2, see Figure 4b, are close to $F_{\mathrm{incoh}}\left(B\to B_{c,2}-\right)\approx 0.56$. Similar observation holds for all studied values of B⩽0.5T, see Figure 4d; a typical deviation does not exceed 5%.

### 4.2. Smooth Potential Barriers

In this subsection, we extend our numerical analysis to smooth potential barriers, defined by choosing 2⩽m<∞ in Equation (2). Moreover, the barrier height is now finite, i.e., $V_{0}=t_{0}/2=1.35\;$eV, being not far from the results of some first-principles calculations for graphene–metal structures [67,68]. To the best of our knowledge, such a model, first proposed in Ref. [40], seems to be the simplest, providing a qualitatively correct description of the conductance spectrum asymmetry observed in existing experiments [46,48,51,54], in which the conductance for μ<0 is noticeably suppressed compared with the μ>0 range, due to the presence of two circular p-n junctions in the former case. (Such a feature is also correctly reproduced by a simpler model assuming the trapezoidal potential barrier [69], which allows a fully analytical treatment, but this approach produces an artificial conductance maximum near μ=0).

**Figure 5:**
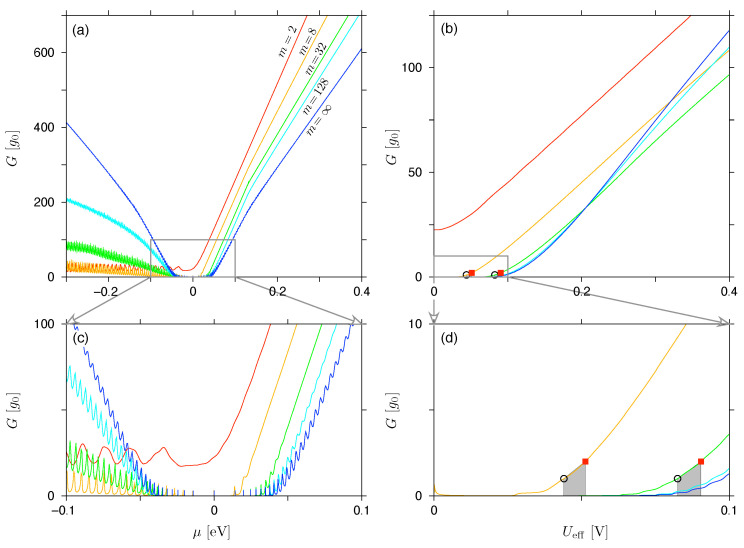
(**a**) Linear-response conductance (*G*) as a function of the chemical potential (μ) and (**b**) finite-voltage conductance, for $\mu =-eU_{\mathrm{eff}}/2$, as a function of the voltage. The magnetic field is B=0.2T for all plots. The disk radii are the same as in Figure 3, but the barrier height, see Equation (2), is now fixed at $V_{0}=t_{0}/2=1.35\;$eV; the parameter *m* is specified for each line. (**c**,**d**) Zoom-in, for low energies, with same datasets as in (**a**,**b**). Datapoints in (**b**,**d**) mark the values of $G\left(U_{\mathrm{on}}^{\left(i\right)}\right)=ig_{0}$, i=1,2, defining the activation voltages $U_{\mathrm{eff}}=U_{\mathrm{on}}^{\left(i\right)}$.

The conductance spectra for five selected values of *m* are displayed in Figure 5, both for the linear-response regime (see Figure 5a,c) and beyond (Figure 5b,d). This time, we have limited our presentation to a single value of the magnetic field, i.e., B=0.2T. It should be noted that a finite value of $V_{0}$ leads to a small but visible asymmetry of the spectrum, even for m=∞.

The finite-voltage results, $G(U_{\mathrm{eff}})$ at $\mu =-eU_{\mathrm{eff}}/2$, allow us to determine the activation voltage, $U_{\mathrm{on}}\left(B\right)$, in a similar manner as for the infinite barrier case (see previous subsection). When attempting to apply the incoherent-scattering approximation to smooth potentials, some modification is required for Equation (46), which now can be rewritten as

(48) $$U_{\mathrm{on},\mathrm{incoh}}=v_{F}BL_{\mathrm{diff}}\left(m\right).$$

In the above, we have introduced the *m*-dependent effective sample length given by [40,41]

(49) $$L_{\mathrm{diff}}\left(m\right)=\left|R_{o}-R_{i}\right|{\left(\frac{\hbar v_{F}}{|R_{o}-R_{i}|V_{0}}\right)}^{1/m},$$

which reduces to $L_{\mathrm{diff}}\left(\infty \right)=R_{o}-R_{i}$ for a rectangular barrier, and gives $L_{\mathrm{diff}}\left(m=2\right)\ll R_{o}-R_{i}$ for the parabolic case. In brief, Equation (49) can be derived from $V\left(\pm L_{\mathrm{diff}}/2\right)=-E_{\mathrm{diff}}$, where $E_{\mathrm{diff}}$ denotes the value of the Fermi energy above which the Sharvin conductance overrules the pseudodiffusive conductance, namely,

(50) $$E_{\mathrm{diff}}=\frac{\hbar v_{F}}{R_{o}-R_{i}}\approx 1\;\mathrm{meV}\;\;\;\mathrm{for}\;\;\;R_{o}-R_{i}=500\;\mathrm{nm}.$$

**Figure 6:**
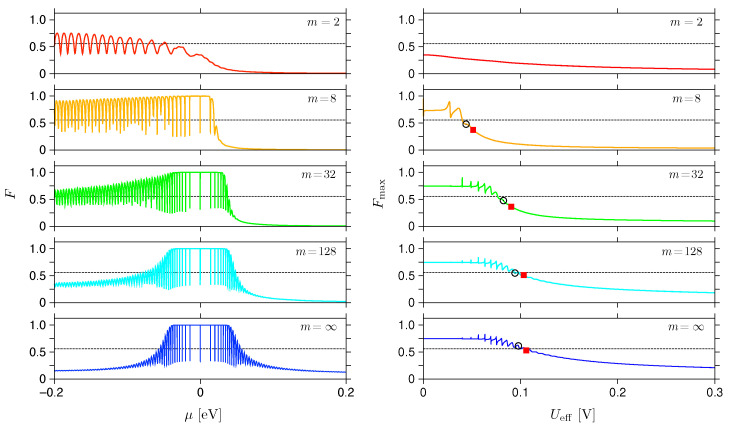
(**Left**): linear-response Fano factor as a function of the chemical potential. (**Right**): finite-voltage Fano factor, for $\mu =-eU_{\mathrm{eff}}/2$, as a function of the voltage. The magnetic field is B=0.2T for all plots, the value of exponent *m* is specified at each plot, and remaining parameters are same as in Figure 5. Horizontal line at each plot marks the value of $F_{\mathrm{incoh}}\left(B\to B_{c,2}-\right)=0.557898$, see Table 1. Datapoints (**right**) mark the values of $F(U_{\mathrm{on}}^{\left(i\right)})$, i=1,2, corresponding to activation voltages $U_{\mathrm{eff}}=U_{\mathrm{on}}^{\left(i\right)}$, for which $G\left(U_{\mathrm{on}}^{\left(i\right)}\right)=ig_{0}$ (see also Figure 5).

In Figure 6 we show the Fano factor, for the same five values of *m* as previously used for the conductance (see Figure 5) and B=0.2T, as a function μ in the linear-response limit ($U_{\mathrm{eff}}\to 0$), as well as a function $U_{\mathrm{eff}}$ for $\mu =-U_{\mathrm{eff}}/2$ (see left or right side of Figure 6, respectively). Again, the aperiodic oscillations almost vanish when entering the nonlinear response regime; in fact, the shape of $F_{\max}\left(U_{\mathrm{eff}}\right)$ appears to be much less sensitive to the value of *m* than the linear-response, F(μ). The datapoints on the right side of Figure 6, identifying the values of $F(U_{\mathrm{on}}^{\left(i\right)})$, i=1,2, such that $G\left(U_{\mathrm{on}}^{\left(i\right)}\right)=ig_{0}$ (see Figure 5), are available starting from m=8 (although the deviation from $F_{\mathrm{incoh}}\left(B\to B_{c,2}-\right)\approx 0.56$ is significant in such a case), whereas strong asymmetry of F(μ) is visible up to m=32.

**Figure 7:**
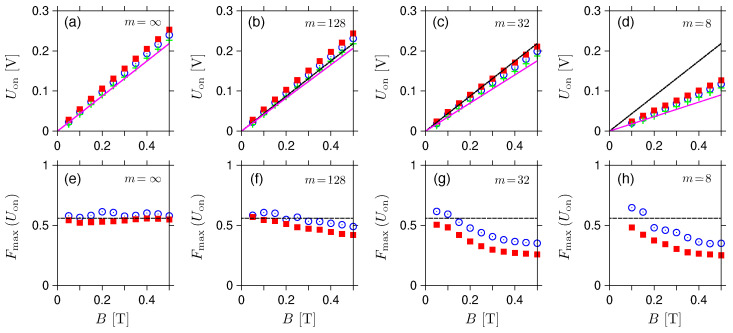
(**a**–**d**) The activation voltage (for the definition, see Figure 4) and (**e**–**h**) the corresponding Fano factor for $\mu =-eU_{\mathrm{eff}}/2$, displayed as functions of the magnetic field (datapoints). The value of exponent *m* is specified at each plot; remaining parameters are same as in Figure 5. Lines in (**a**–**d**) depict the approximation given by Equation (48) (purple solid) and Equation (46) (black dashed) coinciding in the m→∞ limit. Horizontal lines in (**e**–**h**) mark the value of $F_{\mathrm{incoh}}\left(B\to B_{c,2}-\right)=0.557898$, see Table 1.

The values of $U_{\mathrm{on}}^{\left(i\right)}$ and the corresponding $F(U_{\mathrm{on}}^{\left(i\right)})$, for the magnetic fields up to B⩽0.5T, are displayed in Figure 7. It can be noticed that the voltages, $U_{\mathrm{on}}^{\left(i\right)}$, see datapoints in Figure 7a–d, show relatively good agreement with the approximation given by Equation (48) (purple solid lines); in fact, significant deviation from Equation (46) relevant for the rectangular barrier (black dashed lines) can be observed only for m=8. On the contrary, the corresponding Fano factors, $F(U_{\mathrm{on}}^{\left(i\right)})$, see datapoints in Figure 7e–h, remain close to the value of $F_{\mathrm{incoh}}\left(B\to B_{c,2}-\right)\approx 0.56$ only for m=∞ and m=128, showing that the incoherent treatment of the shot-noise power that we propose in Section 3 is applicable only when the potential profiles are close to (but not necessarily perfectly matching) the rectangular shape.

## 5. Conclusions

We have proposed the analytical description of the shot-noise power in graphene-based disks in high magnetic field and doping. Assuming the incoherent scattering of Dirac fermions between two potential steps of an infinite height, both characterized by *a priori* non-zero transmission probability due to the Klein tunneling, we find that the vanishing conductance should be accompanied by the Fano factor F≈0.56, which is weakly dependent on the disk proportions.

Next, the results of analytical considerations are confronted with the outcome of computer simulations, including both rectangular and smooth shapes of the electrostatic potential barrier in the disk area. Calculating both linear-response and finite-voltage transport cumulants, within the zero-temperature Landauer–Büttiker formalism, we point out that the role of evanescent waves (earlier ignored in the analytic approach) is significant in the linear-response regime, but one should be able to detect the quasi-universal F≈0.56 noise in a properly designed experiment going beyond the linear-response regime. To achieve this goal, the following procedure is suggested. First, the activation voltage (for a fixed magnetic field) must be determined, by finding a cusp position on the conductance-versus-voltage plot, above which the conductance grows rapidly with voltage (the average chemical potential is controlled by the gate so that the conductance is minimal for a given voltage). Having determined the activation voltage, one measures the noise for such a voltage, expecting the Fano factor to be close to F≈0.56.

We expect that the effect we describe should be observable in ultraclean samples and at sub-kelvin temperatures (such as in Ref. [49]); at higher temperatures, hydrodynamic effects may noticeably alter the measurable quantities [54]. Since the noise-related characteristics seem to be generally more sensitive to the potential shape than the conductance (or the thermoelectric properties discussed earlier in Ref. [55]), the experimental study following the scenario presented here may be a suitable way to check whether the flat-potential area of a mesoscopic size is present or not in a given graphene-based structure.

Although the work focuses primarily on graphene, recent progress in the fabrication of semiconductor artificial graphenes [70,71] suggests that our results may also be relevant to such systems.

## Figures and Tables

**Table 1:** Selected numerical values of $F_{\mathrm{incoh}}\left(B\;\to \;0\right)$, see Equation (36), and $F_{\mathrm{incoh}}\left(B\;\to \;B_{c,2}-\right)$, see Equation (44). Box marks the values for a=0.5 (i.e., $R_{o}=2R_{i}$) to be compared with the results following from numerical simulations of quantum transport presented Section 4.

$\;a=R_{i}/R_{o}\;$	$\;F_{\mathrm{incoh}}\left(B\to 0\right)\;$	$\;F_{\mathrm{incoh}}\left(B\to B_{c,2}-\right)\;$
0	0.106528	1
0.1	0.106705	0.630994
0.2	0.107239	0.591829
0.3	0.108136	0.573885
0.4	0.109409	0.563905
0.5	0.111074	0.557898
0.6	0.113151	0.554178
0.7	0.115663	0.551894
0.8	0.118619	0.550565
0.9	0.121963	0.549899
1.0	0.125000	0.549708

## Data Availability

Data are contained within the article.

## Appendix A. Numerical Mode Matching for Smooth Potentials

Here we summarize the numerical approach presented earlier in Ref. [55].

In a typical situation, the system of ordinary differential equations for the spinor components $\left(\chi_{a},\chi_{b}\right)$, see Equations (4) and (5), needs to be integrated numerically for all *j*-s. In order to reduce round-off errors that can occur in finite-precision arithmetic due to exponentially growing (or decaying) solutions, one can divide the entire interval $R_{i}<r<R_{o}$ into *M* parts bounded by

(A1) $$\begin{array}{cc} R_{c}^{\left(l\right)} & =R_{i}+l\frac{R_{o}-R_{i}}{M}<r<R_{c}^{\left(l+1\right)},\\ \; & \mathrm{with}\;\;\;l=0,1,\ldots ,M-1. \end{array}$$

(In particular, $R_{c}^{\left(0\right)}=R_{i}$ and $R_{c}^{\left(M\right)}=R_{o}$).

The wave function in the disk area $\chi_{j}^{\left(\mathrm{disk}\right)}$ is now given by the set of functions $\left\{\chi_{j}^{\left(l\right)}\right\}$ for *M* consecutive intervals given by Equation (A1). For the *l*-th interval,

(A2) $$\chi_{j}^{\left(l\right)}=A_{j}^{\left(l\right)}\chi_{j}^{(l),I}+B_{j}^{\left(l\right)}\chi_{j}^{(l),\mathrm{II}},$$

where $\chi_{j}^{(l),I}$, $\chi_{j}^{(l),\mathrm{II}}$ are two linearly independent solutions obtained by integration of Equations (4) and (5) with two different initial conditions, ${\left.\chi_{j}^{(l),I}\right|}_{r=R_{i}^{\left(l\right)}}={\left(1,0\right)}^{T}$ and ${\left.\chi_{j}^{(l),\mathrm{II}}\right|}_{r=R_{i}^{\left(l\right)}}={\left(0,1\right)}^{T}$. $A_{j}^{\left(l\right)}$ and $B_{j}^{\left(l\right)}$ are complex coefficients (to be determined later).

In particular, for $R_{o}=2R_{i}=1000\;$nm and B<0.5T considered in this paper, it is sufficient to set M=20 and employ a standard fourth-order Runge–Kutta (RK4) algorithm with a spatial step of 0.5pm. (For such a choice, the output numerical uncertainties of the transmission probabilities $T_{j}$ are smaller then $10^{-7}$).

The matching conditions for the M+1 interfaces at $r=R_{i}$, $r=R_{c}^{\left(1\right)}$, …, $r=R_{c}^{\left(M-1\right)}$, and $r=R_{o}$, can now be written as

(A3) $$\begin{array}{cc} \chi_{j}^{\left(\mathrm{inner}\right)}\left(R_{i}\right) & =\chi_{j}^{\left(0\right)}\left(R_{i}\right), \end{array}$$

(A4) $$\begin{array}{cc} \chi_{j}^{\left(l\right)}\left(R_{c}^{\left(l+1\right)}\right) & =\chi_{j}^{\left(l+1\right)}\left(R_{c}^{\left(l+1\right)}\right),\;\;\;l=0,\ldots ,M-2, \end{array}$$

(A5) $$\begin{array}{cc} \chi_{j}^{\left(M-1\right)}\left(R_{o}\right) & =\chi_{j}^{\left(\mathrm{outer}\right)}\left(R_{o}\right), \end{array}$$

and are equivalent to the Cramer’s system of 2(M+1) linear equations for the unknowns $A_{j}^{\left(0\right)}$, $B_{j}^{\left(0\right)}$, …, $A_{j}^{\left(M-1\right)}$, $B_{j}^{\left(M-1\right)}$, $r_{j}$, and $t_{j}$.

Writing down explicitly the spinor components appearing in Equations (A3)–(A5), we arrive to

$$\left[\begin{array}{ccccccc} -\chi_{j,a}^{\mathrm{out}}\left(R_{i}\right) & \chi_{j,a}^{(0),I}\left(R_{c}^{\left(0\right)}\right) & \chi_{j,a}^{(0),\mathrm{II}}\left(R_{c}^{\left(0\right)}\right) & & & & \\ -\chi_{j,b}^{\mathrm{out}}\left(R_{i}\right) & \chi_{j,b}^{(0),I}\left(R_{c}^{\left(0\right)}\right) & \chi_{j,b}^{(0),\mathrm{II}}\left(R_{c}^{\left(0\right)}\right) & & & & \\ 0 & \chi_{a}^{(0),I}\left(R_{c}^{\left(1\right)}\right) & \chi_{j,a}^{(0),\mathrm{II}}\left(R_{c}^{\left(1\right)}\right) & & & & \\ 0 & \chi_{b}^{(0),I}\left(R_{c}^{\left(1\right)}\right) & \chi_{j,b}^{(0),\mathrm{II}}\left(R_{c}^{\left(1\right)}\right) & \ddots & & & \\ & & & \ddots & \chi_{j,a}^{(\bar{M}),I}\left(R_{c}^{\left(\bar{M}\right)}\right) & \chi_{j,a}^{(\bar{M}),\mathrm{II}}\left(R_{c}^{\left(\bar{M}\right)}\right) & 0\\ & & & & \chi_{j,b}^{(\bar{M}),I}\left(R_{c}^{\left(\bar{M}\right)}\right) & \chi_{j,b}^{(\bar{M}),\mathrm{II}}\left(R_{c}^{\left(\bar{M}\right)}\right) & 0\\ & & & & \chi_{a}^{(\bar{M}),I}\left(R_{c}^{\left(M\right)}\right) & \chi_{j,a}^{(\bar{M}),\mathrm{II}}\left(R_{c}^{\left(M\right)}\right) & -\chi_{j,a}^{\mathrm{in}}\left(R_{o}\right)\\ & & & & \chi_{b}^{(\bar{M}),I}\left(R_{c}^{\left(M\right)}\right) & \chi_{j,b}^{(\bar{M}),\mathrm{II}}\left(R_{c}^{\left(M\right)}\right) & -\chi_{j,b}^{\mathrm{in}}\left(R_{o}\right) \end{array}\right]$$

(A6) $$\times \;\left[\begin{array}{c} r_{j}\\ A_{j}^{\left(0\right)}\\ B_{j}^{\left(0\right)}\\ \vdots \\ A_{j}^{\left(M-1\right)}\\ B_{j}^{\left(M-1\right)}\\ t_{j} \end{array}\right]=\left[\begin{array}{c} \;\chi_{j,a}^{\mathrm{in}}\left(R_{i}\right)\;\\ \;\chi_{j,b}^{\mathrm{in}}\left(R_{i}\right)\;\\ 0\\ \vdots \\ 0 \end{array}\right],$$

where we have defined $\bar{M}=M-1$,

(A7) $$\chi_{j}^{\mathrm{in}}\;=\left(\begin{array}{c} H_{j-1/2}^{\left(2\right)}\left(Kr\right)\\ iH_{j+1/2}^{\left(2\right)}\left(Kr\right) \end{array}\right),\;\;\;\chi_{j}^{\mathrm{out}}\;=\left(\begin{array}{c} H_{j-1/2}^{\left(1\right)}\left(Kr\right)\\ iH_{j+1/2}^{\left(1\right)}\left(Kr\right) \end{array}\right).$$

For heavily doped leads ($V_{0}\to \infty$), the wave functions given by Equation (A7) simplify to

(A8) $$\chi_{j}^{\left(\mathrm{in}\right)}=\frac{e^{iKr}}{\sqrt{r}}\left(\begin{array}{c} 1\\ 1 \end{array}\right),\;\;\;\;\chi_{j}^{\left(\mathrm{out}\right)}=\frac{e^{-iKr}}{\sqrt{r}}\left(\begin{array}{c} 1\\ -1 \end{array}\right),$$

with $K=|E+V_{0}|/\left(\hbar v_{F}\right)\to \infty$.

As the linear systems for different values of *j*-s are decoupled, standard software packages can be used to find their solutions. We have chosen the double precision LAPACK routine zgesv, see Ref. [72].
